# Analysis of the Safety of Pegfilgrastim Addition in Bleomycin, Etoposide, and Cisplatin Treatment Patients With Germ Cell Tumors

**DOI:** 10.3389/fonc.2021.770067

**Published:** 2022-01-07

**Authors:** Ryunosuke Nakagawa, Hiroaki Iwamoto, Tomoyuki Makino, Suguru Kadomoto, Hiroshi Yaegashi, Masashi Iijima, Shohei Kawaguchi, Takahiro Nohara, Kazuyoshi Shigehara, Kouji Izumi, Yoshifumi Kadono, Atsushi Mizokami

**Affiliations:** ^1^ Department of Integrative Cancer Therapy and Urology, Kanazawa University Graduate School of Medical Science, Kanazawa, Japan; ^2^ Department of Urology, Ishikawa Prefectural Central Hospital, Kanazawa, Japan

**Keywords:** bleomycin, etoposide, and cisplatin (BEP) treatment, germ cell tumor, neutropenia, granulocyte colony-stimulating factor, pegfilgrastim

## Abstract

It has been reported that chemotherapy drugs and granulocyte colony-stimulating factor (G-CSF) administered on the same day can aggravate neutropenia. In the present study, we investigated the safety of pegfilgrastim during bleomycin, etoposide, and cisplatin (BEP) therapy. This single-center retrospective study, including 137 cycles of BEP therapy for germ cell tumors between January 2008 and April 2021, investigated safety. Short-acting G-CSF was used for 84 cycles and pegfilgrastim was used for 53 cycles. In the pegfilgrastim group, neutrophil count at nadir was significantly higher than in the G-CSF group (median 1,650/μl and 680/μl, respectively). The incidence of grade 3–4 neutropenia was significantly higher and the duration longer in the G-CSF group. Also, there was no significant difference in the incidence of febrile neutropenia. In conclusion, concomitant use of pegfilgrastim during BEP therapy did not increase neutropenia and was effective in terms of safety.

## Introduction

Bleomycin, etoposide, and cisplatin (BEP) chemotherapy has been positioned as the standard of care of initial treatment for advanced germ cell tumors (GCT). The most serious side effects of BEP therapy are neutropenia and febrile neutropenia (FN). The incidence of Common Terminology Criteria for Adverse Events (CTCAE) grade 3–4 neutropenia is as high as 73% (1). In addition, these adverse effects of BEP therapy are reported to worsen the prognosis by decreasing the dose intensity and prolonging the dosing interval (2). Furthermore, the use of broad-spectrum antibiotics and prolonged hospitalization in the event of FN increases the cost of treatment and the burden on patients (3). Treatment guidelines recommend the use of granulocyte colony-stimulating factor (G-CSF) prophylaxis to prevent FN in patients deemed to be at high and moderate risk of developing it when patient-related risk factors are added (4, 5). Prophylactic administration of G-CSF is approved for BEP therapy, and there are also long-acting formulations of G-CSF called glycopegylated G-CSF, one of which is pegfilgrastim. By pegylation of filgrastim, the plasma clearance of the renal drug is reduced and the half-life is prolonged, thereby maintaining stable blood concentrations (6). This means that G-CSF needs to be administered every day, while pegfilgrastim only needs to be administered once a cycle. There are several reports comparing the use of pegfilgrastim with filgrastim during chemotherapy (7–11) (**Table 1**). These studies show that the combination of pegfilgrastim is able to reduce severe neutropenia as well as or better than the combination of filgrastim. There are also several reports of a lower incidence of FN than with filgrastim. Cerchione et al. suggest that primary prophylactic use of pegfilgrastim give significant advantages in terms of reduction of chemotherapy disruption due to FN, with subsequent overall improvement of treatment effectiveness. In addition, they noted that the use of pegfilgrastim is highly beneficial for high-risk patients such as the elderly (12). Pegfilgrastim is frequently used prophylactically when anticancer drugs are administered for malignant lymphoma and breast cancer (9, 13). In the urological field, it is administered prophylactically when cabazitaxel is administered for castration-resistant prostate cancer (14). Pegfilgrastim has not been established to be safe when administered 14 days prior to the beginning of chemotherapeutic agents and within 24 hours after the end of chemotherapy. One of the reasons for this is the aggravation of myelosuppression in combination with chemotherapeutic agents (15). In our institution, bleomycin is administered on days 1, 8, and 15, and etoposide and cisplatin on days 1–5. Therefore, bleomycin on days 8 and 15 may be a barrier in BEP therapy when using pegfilgrastim. However, Iwamoto et al. reported that pegfilgrastim can be used safely and effectively during BEP therapy (16). In this study, we accumulated more cases and investigated the safety of adding pegfilgrastim to BEP treatment regimens.

**Table 1:** Studies of prophylactic use of pegfilgrastim versus filgrastim during chemotherapy.

Authors	Cancer type	Regimen	Primary endpoint	Outcome(pegfilgrastim vs. filgrastim)	FN incidence rate (%)(pegfilgrastim vs. filgrastim)
Kubo et al. (7)	malignant lymphoma	cyclophosphamide, cytarabine, etoposide and dexamethasone ± rituximab	number of days with neutrophil count <0.5×10^9^/l in the first cycle	4.5 ± 1.2 days vs. 4.7 ± 1.3 days (p<0.001)	56.6 vs. 55.6
Cerchione et al. (8)	non-Hodgkin lymphoma	bendamustine and rituximab	chemotherapy disruption due to FN	1.6% vs. 11.5% (p=0.028)	27.8 vs. 8.2 (p=0.005)
Xie et al. (9)	breast cancer	epirubicin and cyclophosphamide or epirubicin and docetaxel or docetaxel and cyclophosphamide	incidence and duration of grade 3/4 neutropenia in cycle 1	44.39% vs. 48.45% (NS) 0.96 ± 1.29days vs. 1.10 ± 1.44days (NS)	NS
Green et al. (10)	breast cancer	doxorubicin and docetaxel	duration of grade 4 neutropenia in cycle 1	1.8 ± 1.4days vs. 1.6 ± 1.1 days (p=0.23)	13 vs. 20 (NS)
Holmes et al. (11)	breast cancer	doxorubicin and docetaxel	duration of grade 4 neutropenia in cycle 1	1.7 ± 1.5days vs. 1.8 ± 1.4days (p>0.500)	9 vs. 18 (p=0.029)

FN, febrile neutropenia; NS, not significant.

## Material and Methods

This is a retrospective study. Patients who underwent BEP therapy for germ cell tumors at the Department of Urology of our hospital between January 2008 and April 2021 were included in the study. The BEP regimen consisted of 30 mg of bleomycin on days 1, 8, and 15; 100 mg/m^2^ of etoposide on days 1–5; and 20 mg/m^2^ of cisplatin on days 1–5. Treatment was repeated every three weeks for two to four cycles. Two groups were compared: the group receiving the short-acting G-CSF group (hereafter referred to as short G-CSF), and the pegfilgrastim group. The pegfilgrastim group received 3.6 mg of pegfilgrastim on day 7. The short G-CSF group received filgrastim, lenograstim, or nartograstim and the timing, frequency, and dose were left to the discretion of the attending physician. Age, clinical stage, pathological results, course of treatment, blood sampling data, side effects, and post-hospitalization course were retrospectively investigated. Statistical analyses were performed using the commercially available software Prism 8 (GraphPad, San Diego, CA, USA). Comparisons between different groups were performed using the chi-squared test and the Mann–Whitney U test. In all analyses, p-values of less than 0.05 indicated statistical significance. This study was approved by the institutional review board of Kanazawa University Hospital (2021-041).

## Results

The short G-CSF group had a total of 84 cycles and the pegfilgrastim group had 53 cycles (9 of the 53 cycles were combined with G-CSF). The median age of the patients was 35 years in the short G-CSF group and 42 years in the pegfilgrastim group. There was no significant difference in the primary tumor, pathology, clinical stage, International Germ Cell Consensus Classification (IGCCC), or metastatic site between the two groups (**Table 2**).

**Table 2:** Patient characteristics.

	short G-CSF	pegfilgrastim	p-value
**Cycle**	84	53^※1^	
**No. of patients^※2^**	29	19	
**Age**	n (%)	n (%)	
median	35	42	0.74
range	14–66	18–55	
**primary tumor**			
testis	25 (86)	17 (89)	0.72
anterior mediastinum	3 (10)	2 (11)	
retroperitoneum	1 (4)	0	
**pathology**			
seminoma	13 (44)	8 (42)	0.69
mixed	15 (51)	11 (58)	
unknown	1 (5)	0	
**clinical stage**			0.15
I	0	3 (15)	
II	13 (44)	8 (42)	
III	15 (51)	7 (36)	
unknown	1 (5)	1 (5)	
**IGCCC**			0.30
Good	15 (51)	14 (73)	
Intermediate	8 (27)	4 (21)	
Poor	5 (22)	1 (6)	
**distant metastasis**			0.47
LN	23 (79)	15 (79)	
Lung	7 (24)	4 (21)	
Liver	1 (3)	2 (10)	
Bone	0	2 (10)	
Retroperitoneum	1 (3)	0	
None	3 (10)	3 (15)	

IGCCC, International Germ Cell Consensus Classification.

※1. 9 of 53 cycles were combined with G-CSF.

※2. The total number of patients was 44, of which 4 had overlapping patient numbers as some patients belonged to both groups.

**Table 3** shows hematological toxicity and other events in each group. The nadir of the absolute neutrophil count (ANC) was significantly higher in the pegfilgrastim group than in the short G-CSF group (median 1,650/μl and 680/μl, respectively, p = 0.0045). The maximum neutrophil count (MNC) was also significantly higher in the pegfilgrastim group than in the short G-CSF group (median 15,490/μl and 11,600/μl, respectively, p = 0.0052). There were five cycles of FN in each group, and no significant difference was observed (p = 0.509). Only the pegfilgrastim group was discharged during the cycle. This difference was also significant (0 and 12 cycles, respectively, p < 0.0001). The number of cycles for which bleomycin was discontinued was 7 and 5 cycles, respectively. There was also no significant difference in the incidence of interstitial pneumonia (p = 0.740). As for grade 3–4 serious adverse events, the incidence of neutropenia was significantly higher in the short G-CSF group (p = 0.0014), while there was no significant difference in the incidence of anemia and thrombocytopenia.

**Table 3:** Hematological adverse events and other events.

	short G-CSF	pegfilgrastim	p-Value
**Cycle administered,n**	84	53	
**Median no. of administrations per cycle**	7 (1–14)	1	
**ANC at nadir (10^3^/μl)**	0.68 (0.02–3.31)	1.65 (0.01–8.18)	0.0045
**MNC (10^3^/μl)**	11.6 (4.39–93.7)	15.49 (2.9–85.59)	0.0052
**FN (%)**	5 (5.95)	5 (9.43)	0.51
**Discharge,n**	0	12	<0.0001
**Discontinuation of BLM,n (%)**	7 (8.43)	5 (9.43)	>0.99
** Cause of discontinuation**			
** FN**	3 (3.57)	3 (5.66)	0.56
** Interstitial pneumonia**	1 (1.19)	1 (1.88)	0.74
** Erythroderma with fever**	1 (1.19)	0	0.43
** Fever**	0	1 (1.88)	0.21
** Neutropenia**	2 (2.38)	0	0.26
**Grade 3–4 adverse event**			
**Neutropenia**	57 (67)	21 (39)	0.0014
**Anemia**	4 (4.76)	3 (5.66)	>0.99
**Thrombocytopenia**	0	2 (3.77)	0.15

ANC, absolute neutrophil count; MNC, maximum neutrophil count; FN, febrile neutropenia; BLM, bleomycin.

**Figure 1:**
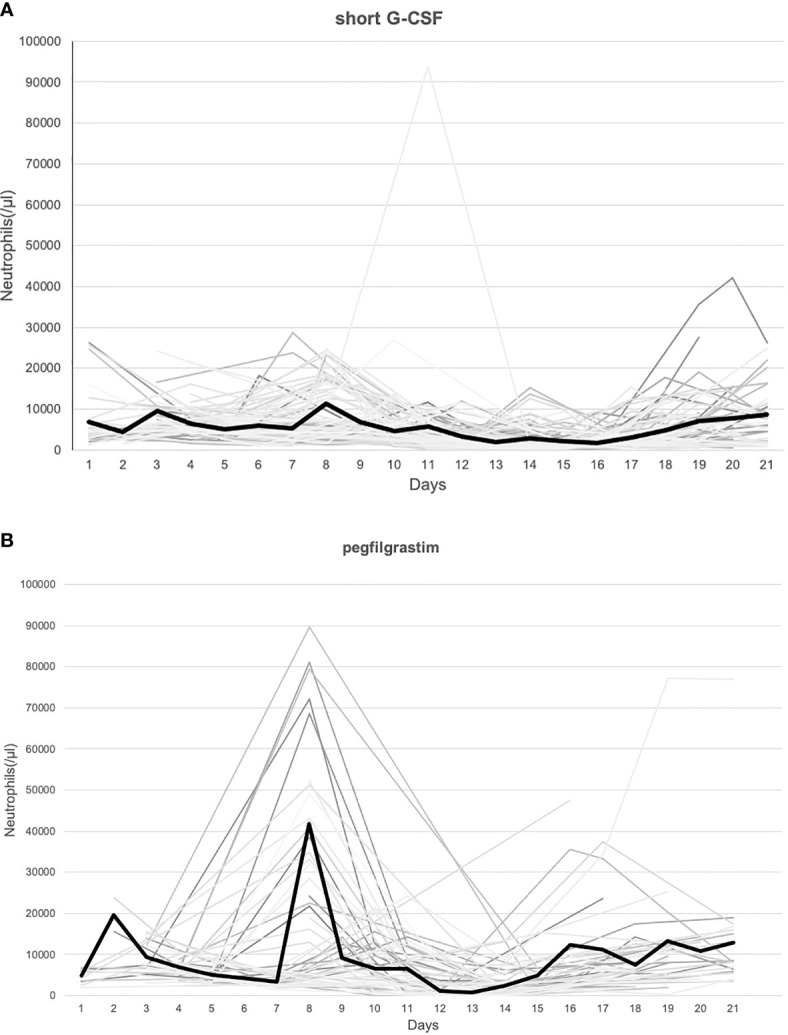
**(A)** The number of neutrophils in all cycles in the short G-CSF group. **(B)** The number of neutrophils in all cycles in the pegfilgrastim group.※ Bold lines indicate the average values each day.

**Figure 2:**
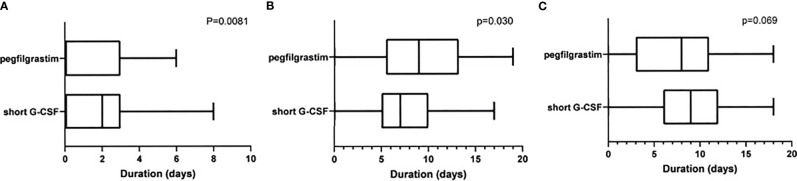
**(A)** Duration of neutropenia (CTCAE grade 3–4). **(B)** Duration of ANC>5000/μl. **(C)** Duration of ANC 1000–5000/μl.

**Figure 1** shows the ANC for each day in each cycle. The bold line shows the average value of ANC. Since pegfilgrastim was administered on day 7, neutrophils in the pegfilgrastim group reached peak on day 8. On the other hand, the number of times and the day of administration of G-CSF were left to the discretion of the physician, leading to variations in the peak of neutrophils. The duration of grade 3–4 neutropenia in the cycle was significantly longer in the short G-CSF group than the pegfilgrastim (p = 0.0081, median of 2 and 0 days, respectively, **Figure 2A**). On the other hand, the duration of ANC > 5,000/μl was significantly longer in the pegfilgrastim group than the short G-CSF group (p = 0.0303, median of 9 and 7 days, respectively, **Figure 2B**), but there was no significant difference in the duration of ANC 1,000–5,000/μl (p = 0.0693, **Figure 2C**).

**Figure 3:**
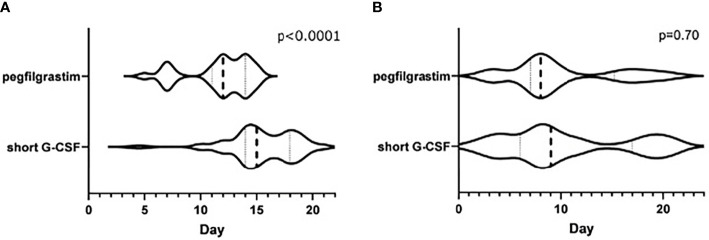
**(A)** Day that demonstrated the ANC at nadir during the cycle. **(B)** Day that demonstrated the MNC during the cycle.

**Figure 3** shows a violin plot of the time points during the cycle that led to ANC at nadir or MNC. There was a significant difference in the median number of days for ANC at nadir (day 12 for the pegfilgrastim group and day 15 for the short G-CSF group, p < 0.0001). Also, in the pegfilgrastim group, nadir ANC was consolidated by day 17, while there were patients in the short G-CSF group who reached nadir after day 17 (**Figure 3A**). MNC was widely distributed in both groups (**Figure 3B**).

## Discussion

According to the National Comprehensive Cancer Network guidelines on the use of myeloid growth factors, pegfilgrastim is recommended to be administered after the day of chemotherapy administration (17). The reason for this is that myeloid progenitor cells stimulated by myeloproliferative factors after the same day administration of G-CSF may be affected by cytotoxic chemotherapy, resulting in neutropenia (18). But there are some reports denying the same day administration (19, 20), and others supporting it (21). Burris et al. reported that in a breast cancer study, the mean severe neutropenia duration was 1.2 days longer in the same day pegfilgrastim compared with the next-day group (20) and this was statistically significant. On the other hand, Schuman et al. demonstrated safety and efficacy of pegfilgrastim given on the same day as myelosuppressive chemotherapy in patients with gynecologic malignancies (21). Lipegfilgrastim, which has not yet been introduced in Japan, has the same effect as pegfilgrastim. Lipegfilgrastim is a glycopegylated G-CSF with a prolonged half-life compared with the same dose of pegfilgrastim and produces a more stable ANC increase (22). A large observational study using lipegfilgrastim in combination with chemotherapy has shown its safety and efficacy. In this study, prophylactic treatment with lipegfilgrastim in 228 patients with urologic cancer resulted in a low incidence of FN (2.6%) and grade 3–4 neutropenia (2.2%). The study also included 50 patients with testicular tumors, 88% of whom received BEP therapy (23).

Pegfilgrastim and 11 days’ filgrastim have similar efficacy and safety (24). In this study, pegfilgrastim was given on day 7, which means that it was given on the same day as bleomycin on days 8 and 15 in BEP therapy. However, despite the administration of a long-acting G-CSF during BEP therapy, the nadir of ANC was significantly higher than in the regular G-CSF group, and there was no significant difference in the incidence of FN. Bleomycin has been reported to cause little or no myelosuppression in *in vitro* experiments (25). Therefore, it can be inferred that the combination with pegfilgrastim did not lead to neutropenia in BEP therapy. In the evaluable patients in the United States, only 7 of 806 patients (0.008%) had significant thrombocytopenia or leukopenia while receiving bleomycin (26).

In this study, the duration of grade 3–4 neutropenia was significantly shorter in the pegfilgrastim group. It suggests that stable neutrophil counts can be maintained by administering pegfilgrastim. The majority of protocols call for filgrastim to be administered until the ANC is greater than 5000/μL (27). Therefore, we checked the duration of ANC > 5,000/μL and found it was longer in patients who received pegfilgrastim. MNC was also significantly higher in the pegfilgrastim group. Although not present in our study, cases of splenic rupture due to excessive mobilization of hematopoietic stem cells into the peripheral blood by G-CSF have been reported (28, 29). A case of splenic rupture has been reported 8 days after pegfilgrastim was administered during chemotherapy for advanced lung cancer. The white blood cell (WBC) count at diagnosis was 48,800/μl (29). Although there is no data on the baseline value for increased risk of splenic rupture, there were cases in this study in which ultrasonography was not performed even though marked neutrophilia was observed, so improvement of clinical management is needed in the future.

Bleomycin is pulmonary toxic, and the incidence of interstitial pneumonia and pulmonary fibrosis is estimated to be about 10% (30). Although it has been reported that the addition of G-CSF may increase the risk of bleomycin-induced interstitial pneumonia, there have been recent conflicting reports (31). Azoulay et al. injected bleomycin and G-CSF into the trachea of rats and reported that acute lung injury and fibrosis were exacerbated by the addition of G-CSF rather than bleomycin alone (32). On the other hand, Laprise-Lachance et al. reported that the risk of pulmonary toxicity associated with the addition of G-CSF was not statistically significant in their case-control study (33). In this study, there were only two cases of interstitial pneumonia in the entire cycle (one each in the short G-CSF and pegfilgrastim groups), and the incidence was low. Regarding the concern of pulmonary toxicity, we believe that the combination of BEP therapy and pegfilgrastim is feasible.

In the present study, only patients of the pegfilgrastim group were temporarily discharged during the cycle (12 of 53 cycles). This may be due to a short half-life in blood of daily G-CSF, which requires frequent administration and monitoring; while the pegfilgrastim group can sustain a stable granulocyte colony-forming stimulus response, leading to a shorter hospital stay.

For ANC at nadir, readings of the short G-CSF group showed a wide distribution, whereas those of the pegfilgrastim group were concentrated around day 17. Based on these results, we believe that timing of the ANC nadir during pegfilgrastim treatment is predictable, allowing temporary discharge during the cycle and reducing the burden on the patient.

There are several limitations in the current study, the first of which being its retrospective nature. A larger, prospective study would further confirm our results. In addition, this study did not examine the antitumor effects of BEP therapy. Sato et al. reported a reduction in neutropenia and leukopenia when using pegfilgrastim in small cell lung cancer patients, leading to longer progression-free survival than the group that did not use pegfilgrastim (34). It is necessary to study the effect of concomitant use of pegfilgrastim in preventing postponement of chemotherapy and, consequently, in antitumor efficacy.

In conclusion, concomitant use of pegfilgrastim during BEP therapy did not increase the incidence of neutropenia or FN compared with the usual G-CSF combination. The discontinuation rate of bleomycin due to interstitial pneumonia was low and the combination was safe in terms of pulmonary toxicity. Also, concomitant use of pegfilgrastim can help physicians predict when neutrophil nadir will be reached and may eliminate the need for frequent administration of G-CSF products and blood collection monitoring. It also allows for temporary discharge, reducing the burden on patients.

## Data Availability Statement

The original contributions presented in the study are included in the article/supplementary material. Further inquiries can be directed to the corresponding author.

## Ethics Statement

The studies involving human participants were reviewed and approved by Medical Ethics Committee of Kanazawa University. Written informed consent for participation was not required for this study in accordance with the national legislation and the institutional requirements.

## Author Contributions

All authors listed have made a substantial, direct, and intellectual contribution to the work, and approved it for publication.

## Conflict of Interest

The authors declare that the research was conducted in the absence of any commercial or financial relationships that could be construed as a potential conflict of interest.

## Publisher’s Note

All claims expressed in this article are solely those of the authors and do not necessarily represent those of their affiliated organizations, or those of the publisher, the editors and the reviewers. Any product that may be evaluated in this article, or claim that may be made by its manufacturer, is not guaranteed or endorsed by the publisher.

## References

[1] Loehrer PJSR Gonin R Nichols CR Weathers T Einhorn LH. Vinblastine Plus Ifosfamide Plus Cisplatin as Initial Salvage Therapy in Recurrent Germ Cell Tumor. J Clin Oncol (1998) 16(7):2500–4. doi: 10.1200/jco.1998.16.7.2500 9667270

[2] Toner GC Stockler MR Boyer MJ Jones M Thomson DB Harvey VJ. Comparison of Two Standard Chemotherapy Regimens for Good-Prognosis Germ-Cell Tumours: A Randomised Trial. Australian and New Zealand Germ Cell Trial Group. Lancet (2001) 357(9258):739–45. doi: 10.1016/s0140-6736(00)04165-9 11253966

[3] Crawford J Ozer H Stoller R Johnson D Lyman G Tabbara I. Reduction by Granulocyte Colony-Stimulating Factor of Fever and Neutropenia Induced by Chemotherapy in Patients With Small-Cell Lung Cancer. N Engl J Med (1991) 325(3):164–70. doi: 10.1056/nejm199107183250305 1711156

[4] Aapro MS Bohlius J Cameron DA Dal Lago L Donnelly JP Kearney N. 2010 Update of EORTC Guidelines for the Use of Granulocyte-Colony Stimulating Factor to Reduce the Incidence of Chemotherapy-Induced Febrile Neutropenia in Adult Patients With Lymphoproliferative Disorders and Solid Tumours. Eur J Cancer (2011) 47(1):8–32. doi: 10.1016/j.ejca.2010.10.013 21095116

[5] Crawford J Caserta C Roila F. Hematopoietic Growth Factors: ESMO Clinical Practice Guidelines for the Applications. Ann Oncol (2010) 21(Suppl 5):v248–51. doi: 10.1093/annonc/mdq195 20555091

[6] Yang BB Kido A. Pharmacokinetics and Pharmacodynamics of Pegfilgrastim. Clin Pharmacokinet (2011) 50(5):295–306. doi: 10.2165/11586040-000000000-00000 21456630

[7] Kubo K Miyazaki Y Murayama T Shimazaki R Usui N Urabe A. A Randomized, Double-Blind Trial of Pegfilgrastim Versus Filgrastim for the Management of Neutropenia During CHASE(R) Chemotherapy for Malignant Lymphoma. Br J Haematol (2016) 174(4):563–70. doi: 10.1111/bjh.14088 PMC5074273 27072050

[8] Cerchione C De Renzo A Di Perna M Della Pepa R Pugliese N Catalano L. Pegfilgrastim in Primary Prophylaxis of Febrile Neutropenia Following Frontline Bendamustine Plus Rituximab Treatment in Patients With Indolent Non-Hodgkin Lymphoma: A Single Center, Real-Life Experience. Support Care Cancer (2017) 25(3):839–45. doi: 10.1007/s00520-016-3468-8 PMC5266775 27812763

[9] Xie J Cao J Wang JF Zhang BH Zeng XH Zheng H. Advantages With Prophylactic PEG-rhG-CSF Versus rhG-CSF in Breast Cancer Patients Receiving Multiple Cycles of Myelosuppressive Chemotherapy: An Open-Label, Randomized, Multicenter Phase III Study. Breast Cancer Res Treat (2018) 168(2):389–99. doi: 10.1007/s10549-017-4609-6 29230663

[10] Green MD Koelbl H Baselga J Galid A Guillem V Gascon P. A Randomized Double-Blind Multicenter Phase III Study of Fixed-Dose Single-Administration Pegfilgrastim Versus Daily Filgrastim in Patients Receiving Myelosuppressive Chemotherapy. Ann Oncol (2003) 14(1):29–35. doi: 10.1093/annonc/mdg019 12488289

[11] Holmes FA O'Shaughnessy JA Vukelja S Jones SE Shogan J Savin M. Blinded, Randomized, Multicenter Study to Evaluate Single Administration Pegfilgrastim Once Per Cycle Versus Daily Filgrastim as an Adjunct to Chemotherapy in Patients With High-Risk Stage II or Stage III/IV Breast Cancer. J Clin Oncol (2002) 20(3):727–31. doi: 10.1200/jco.2002.20.3.727 11821454

[12] Cerchione C De Renzo A Nappi D Di Perna M Della Pepa R Pugliese N. Pegfilgrastim in Primary Prophylaxis of Febrile Neutropenia in Elderly Patients With Hematological Malignancies-Bendamustine and G-CSF Support. Support Care Cancer (2019) 27(5):1587–8. doi: 10.1007/s00520-019-4651-5 30671660

[13] Fietz T Lück A Schulz H Harde J Losem C Grebhardt S. Prophylaxis of Chemotherapy-Induced Neutropenia and Febrile Neutropenia With Lipegfilgrastim in 2489 Cancer Patients: Final Results From the Non-Interventional Study NADIR. Curr Med Res Opin (2019) 35(7):1127–38. doi: 10.1080/03007995.2018.1560200 30557099

[14] Iwamoto H Kano H Shimada T Naito R Makino T Kadomoto S. Sarcopenia and Visceral Metastasis at Cabazitaxel Initiation Predict Prognosis in Patients With Castration-Resistant Prostate Cancer Receiving Cabazitaxel Chemotherapy. In Vivo (2021) 35(3):1703–9. doi: 10.21873/invivo.12430 PMC8193302 33910855

[15] Li Y Klippel Z Shih X Wang H Reiner M Page JH. Trajectory of Absolute Neutrophil Counts in Patients Treated With Pegfilgrastim on the Day of Chemotherapy Versus the Day After Chemotherapy. Cancer Chemother Pharmacol (2016) 77(4):703–12. doi: 10.1007/s00280-016-2970-5 PMC4819939 26886017

[16] Iwamoto H Izumi K Natsagdorj A Makino T Nohara T Shigehara K. Effectiveness and Safety of Pegfilgrastim in BEP Treatment for Patients With Germ Cell Tumor. In Vivo (2018) 32(4):899–903. doi: 10.21873/invivo.11326 29936477 PMC6117791

[17] Crawford J Becker PS Armitage JO Blayney DW Chavez J Curtin P. Myeloid Growth Factors, Version 2.2017, NCCN Clinical Practice Guidelines in Oncology. J Natl Compr Canc Netw (2017) 15(12):1520–41. doi: 10.6004/jnccn.2017.0175 29223990

[18] Meropol NJ Miller LL Korn EL Braitman LE MacDermott ML Schuchter LM. Severe Myelosuppression Resulting From Concurrent Administration of Granulocyte Colony-Stimulating Factor and Cytotoxic Chemotherapy. J Natl Cancer Inst (1992) 84(15):1201–3. doi: 10.1093/jnci/84.15.1201 1378905

[19] Weycker D Li X Figueredo J Barron R Tzivelekis S Hagiwara M. Risk of Chemotherapy-Induced Febrile Neutropenia in Cancer Patients Receiving Pegfilgrastim Prophylaxis: Does Timing of Administration Matter? Support Care Cancer (2016) 24(5):2309–16. doi: 10.1007/s00520-015-3036-7 PMC4805705 26607482

[20] Burris HA Belani CP Kaufman PA Gordon AN Schwartzberg LS Paroly WS. Pegfilgrastim on the Same Day Versus Next Day of Chemotherapy in Patients With Breast Cancer, Non-Small-Cell Lung Cancer, Ovarian Cancer, and Non-Hodgkin's Lymphoma: Results of Four Multicenter, Double-Blind, Randomized Phase II Studies. J Oncol Pract (2010) 6(3):133–40. doi: 10.1200/jop.091094 PMC2868638 20808556

[21] Schuman SI Lambrou N Robson K Glück S Myriounis N Pearson JM. Pegfilgrastim Dosing on Same Day as Myelosuppressive Chemotherapy for Ovarian or Primary Peritoneal Cancer. J Support Oncol (2009) 7(6):225–8. 20380330

[22] Abdolzade-Bavil A von Kerczek A Cooksey BA Kaufman T Krasney PA Pukac L. Differential Sensitivity of Lipegfilgrastim and Pegfilgrastim to Neutrophil Elastase Correlates With Differences in Clinical Pharmacokinetic Profile. J Clin Pharmacol (2016) 56(2):186–94. doi: 10.1002/jcph.578 26105553

[23] Merseburger AS Geiges G Klier J Wiesholzer M Pichler P. Pooled Analysis on the Effectiveness and Safety of Lipegfilgrastim in Patients With Urological Malignancies in the Real-World Setting. Front Oncol (2021) 11:655355. doi: 10.3389/fonc.2021.655355 34123810 PMC8195268

[24] Aapro M Boccia R Leonard R Camps C Campone M Choquet S. Refining the Role of Pegfilgrastim (a Long-Acting G-CSF) for Prevention of Chemotherapy-Induced Febrile Neutropenia: Consensus Guidance Recommendations. Support Care Cancer (2017) 25(11):3295–304. doi: 10.1007/s00520-017-3842-1 PMC5610660 28842778

[25] Tisman G Herbert V Go LT Brenner L. Marked Immunosuppression With Minimal Myelosuppression by Bleomycin *In Vitro* . Blood (1973) 41(5):721–6. doi: 10.1182/blood.V41.5.721.721 4121004

[26] Blum RH Carter SK Agre K. A Clinical Review of Bleomycin–a New Antineoplastic Agent. Cancer (1973) 31(4):903–14. doi: 10.1002/1097-0142(197304)31:4<903::aid-cncr2820310422>3.0.co;2-n 4122362

[27] Yanada M Takeuchi J Sugiura I Akiyama H Usui N Yagasaki F. High Complete Remission Rate and Promising Outcome by Combination of Imatinib and Chemotherapy for Newly Diagnosed BCR-ABL-Positive Acute Lymphoblastic Leukemia: A Phase II Study by the Japan Adult Leukemia Study Group. J Clin Oncol (2006) 24(3):460–6. doi: 10.1200/jco.2005.03.2177 16344315

[28] Veerappan R Morrison M Williams S Variakojis D. Splenic Rupture in a Patient With Plasma Cell Myeloma Following G-CSF/GM-CSF Administration for Stem Cell Transplantation and Review of the Literature. Bone Marrow Transplant (2007) 40(4):361–4. doi: 10.1038/sj.bmt.1705736 17563733

[29] Benguerfi S Thepault F Lena H Ricordel C. Spontaneous Splenic Rupture as a Rare Complication of G-CSF Injection. BMJ Case Rep (2018) 2018. doi: 10.1136/bcr-2017-222561 PMC5780587 29330272

[30] O'Sullivan JM Huddart RA Norman AR Nicholls J Dearnaley DP Horwich A. Predicting the Risk of Bleomycin Lung Toxicity in Patients With Germ-Cell Tumours. Ann Oncol (2003) 14(1):91–6. doi: 10.1093/annonc/mdg020 12488299

[31] Maruyama Y Sadahira T Mitsui Y Araki M Wada K Tanimoto R. Prognostic Impact of Bleomycin Pulmonary Toxicity on the Outcomes of Patients With Germ Cell Tumors. Med Oncol (2018) 35(6):80. doi: 10.1007/s12032-018-1140-5 29700638

[32] Azoulay E Herigault S Levame M Brochard L Schlemmer B Harf A. Effect of Granulocyte Colony-Stimulating Factor on Bleomycin-Induced Acute Lung Injury and Pulmonary Fibrosis. Crit Care Med (2003) 31(5):1442–8. doi: 10.1097/01.Ccm.0000050453.28177.33 12771616

[33] Laprise-Lachance M Lemieux P Grégoire JP. Risk of Pulmonary Toxicity of Bleomycin and Filgrastim. J Oncol Pharm Pract (2019) 25(7):1638–44. doi: 10.1177/1078155218804293 30319063

[34] Sato Y Iihara H Kinomura M Hirose C Fujii H Endo J. Primary Prophylaxis of Febrile Neutropenia With Pegfilgrastim in Small-Cell Lung Cancer Patients Receiving Amrubicin as Second-Line Therapy. Anticancer Res (2021) 41(3):1615–20. doi: 10.21873/anticanres.14923 33788757

